# Particles Emission from an Industrial Spray Coating Process Using Nano-Materials

**DOI:** 10.3390/nano12030313

**Published:** 2022-01-18

**Authors:** Benedetta Del Secco, Sara Trabucco, Fabrizio Ravegnani, Antti Joonas Koivisto, Ilaria Zanoni, Magda Blosi, Simona Ortelli, Marko Altin, Gianni Bartolini, Anna Luisa Costa, Franco Belosi

**Affiliations:** 1 CNR-ISAC, Institute of Atmospheric Sciences and Climate-National Research Council of Italy, Via Gobetti, 101, 40129 Bologna, Italy; s.trabucco@isac.cnr.it (S.T.); f.ravegnani@isac.cnr.it (F.R.); f.belosi@isac.cnr.it (F.B.); 2 Air Pollution Management APM, Mattilanmäki 38, 33610 Tampere, Finland; joonas.apm@gmail.com; 3 CNR-ISTEC, Institute of Science and Technology for Ceramics-National Research Council of Italy, Via Granarolo 64, 48018 Faenza, Italy; ilaria.zanoni@istec.cnr.it (I.Z.); magda.blosi@istec.cnr.it (M.B.); simona.ortelli@istec.cnr.it (S.O.); anna.costa@istec.cnr.it (A.L.C.); 4 Witek srl., Via Siena 47, 50142 Firenze, Italy; marko.altin@witekgroup.com (M.A.); gianni.bartolini@witekgroup.com (G.B.)

**Keywords:** aerosol, spray coating, nanoparticles, worker exposure

## Abstract

Industrial spray coating processes are known to produce excellent coatings on large surfaces and are thus often used for in-line production. However, they could be one of the most critical sources of worker exposure to ultrafine particles (UFPs). A monitoring campaign at the Witek s.r.l. (Florence, Italy) was deployed to characterize the release of TiO_2_ NPs doped with nitrogen (TiO_2_-N) and Ag capped with hydroxyethyl cellulose (AgHEC) during automatic industrial spray-coating of polymethyl methacrylate (PMMA) and polyester. Aerosol particles were characterized inside the spray chamber at near field (NF) and far field (FF) locations using on-line and off-line instruments. Results showed that TiO_2_-N suspension produced higher particle number concentrations than AgHEC in the size range 0.3–1 µm (on average 1.9 10^2^ p/cm^3^ and 2.5 10^1^ p/cm^3^, respectively) after background removing. At FF, especially at worst case scenario (4 nozzles, 800 mL/min flow rate) for TiO_2_-N, the spray spikes were correlated with NF, with an observed time lag of 1 minute corresponding to a diffusion speed of 0.1 m/s. The averaged ratio between particles mass concentrations in the NF position and inside the spray chamber was 1.7% and 1.5% for TiO_2_-N and for AgHEC suspensions, respectively. The released particles’ number concentration of TiO_2_-N in the size particles range 0.3–1 µm was comparable for both PMMA and polyester substrates, about 1.5 and 1.6 10^2^ p/cm^3^. In the size range 0.01–30 µm, the aerosol number concentration at NF for both suspensions was lower than the nano reference values (NRVs) of 16·10^3^ p/cm^-3^.

## 1. Introduction

Industrial processes are increasingly focused on nanotechnologies and engineered nanomaterials (NMs). Although nanotechnology provides enormous benefits, there is growing alarm about the potential health hazard (particularly for workers) [1] and possible environment damage associated with exposure to NMs, especially ultrafine particles (UFPs) of less than about 0.1 μm [2], given their significantly higher inflammatory potential than fine particles (FPs; over 100 nm) [3]. The biological activity of UFP is due to their huge surface area [4], which induces severe respiratory symptoms leading to decreased lung function and exacerbation of asthma [5,6,7].

Spray-coating is a well-known industrial technique consisting of depositing suspensions of various nanoparticles (NPs) to coat a wide variety of different shaped materials [8,9]. Atomized droplets containing NPs are deposited on the surface, leaving a nanostructured coating once the liquid solvent has evaporated. Compared to other techniques, spray coating presents numerous advantages. These include minimal liquid waste, easily controlled film thickness and roughness, and the possibility of using a broad spectrum of different viscosity fluids. Simple to operate, spray coating can be readily designed into large computer-controlled production systems [10]. Coating quality and potential occupational exposure are strongly dependent on the NMs employed, the characteristics of the dispersing matrix, and process parameters, including spray rate, atomization air pressure, inlet and exhaust air temperature, nozzle size, nozzle-to-bed distance and on-site control measures [11].

Despite the increasing attention given to managing risks in the nanotechnology industry [12], very few occupational exposure studies considering NM release and worker inhalation exposure have been conducted in nanoparticles spraying. In 2017, Ding et al. [13] reviewed studies on the release of engineered nanomaterials (ENMs) in a range of industrial settings, such as flame spray-coating, chemical vapor deposition, gas phase condensation, and normal mixing processes (but not, however, the process examined in our study). The authors confirmed that spraying processes caused high releases of submicron particles, reporting particle number concentrations ranging from approximately 5.7 ·10^5^ to 8·10^6^ p/cm^3^ for ZnO in flame spraying and from 1.2·10^5^ to 2.0·10^5^ for SiO_2_ with the compressor sprayer. Particle size range was 14–673 nm and 10–300 nm, respectively. Thermal spraying in the ceramic industry was found to increase the work area concentrations between ca. 10^4^ to 8.3·10^5^ p/cm^3^ [14,15]. Five trials each lasting 12–15 min resulted in a mean personal exposure during airless spray painting of TiO_2_ particles of 0.7 mg/m^3^ [16]. Koivisto et al. [17] observed mean particle emission rates of 1.9·10^10^ s^-1^ (381 μg/s) in the case of hand-held electrostatic spray-coating. Recently, Ortelli et al. [18] measured particle number concentrations of up to 1.2·10^3^ p/cm^3^ over a particle size range 0.3–1µm in a worker-occupied area in an industrial setting. These studies testify to considerable potential worker exposure risk related to atmospheric nano-coating spraying processes and suggest the need for deeper investigation not only to assess risk but also to evaluate the effectiveness of emission controls, such as spray booths or automated spraying systems. Additionally, spray process involves high air volume flow that is used to atomize the coating suspension. This needs to be taken into account when applying local exhaust ventilation (LEV) controls that can be affected by air and stream direction change when hitting the target. Therefore, a proper design of the spray cabin LEV can reduce the emissions and the use of personal protective equipment (PPEs) can reduce the workers’ exposure risks in spray processes. Various strategies have been developed to asses exposure to NMs in the workplace, mixing different aerosol measurement instruments and considering multiple characteristics that may influence NM toxicity [19,20,21,22]. As part of the European funded ASINA project (GA 862444), a field campaign to monitor a spray coating process for the production of self-cleaning/self-purifying polyester and plastic surfaces was implemented at Wiva Group srl (now Witek srl, Florence, Italy), an advanced technology lighting company and project partner. This paper presents our NP release findings at the industrial spray coating plant for the various materials, processes and substrates.

## 2. Materials and Methods

### 2.1. Materials and Coating Process

The following two NPs suspensions were used in the spray nozzles: TiO_2_-N (1% *w*/*w*) dispersed in EtOH (solution 96% grade solvents, VMR international) and Ag (Sigma Aldrich, Milan, Italy) capped with hydroxyethylcellulose (Univar Solutions SpA, Milan, Italy) (AgHEC), dispersed in water at concentrations of 0.1%, 0.05% and 0.01% *w*/*w*. Specifically, the TiO_2_-N suspension was prepared by Colorobbia Italia, SPA (Sovigliana Vinci, FI, Italy) while the AgHEC aqueous nano suspensions were produced by CNR-ISTEC (Faenza, Italy) using a patented production process [23].

**Figure 1:**
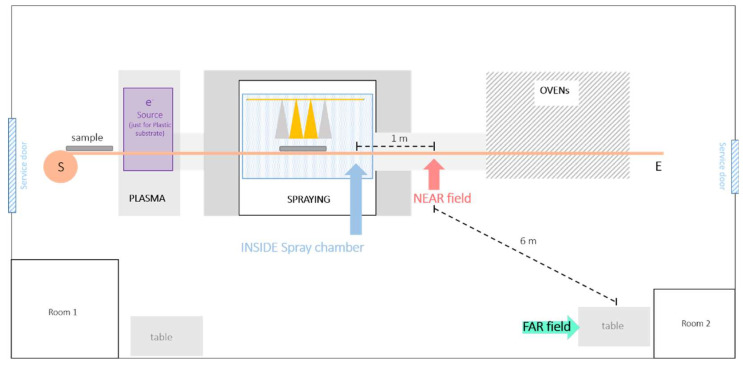
Schematization of the Witek s.r.l plant and measurement stations (inside, near field and far field).

The automatic spray coating is conveyor belt-operated, the substrate passing through a plasma neutralizer to the spray chamber and then to a drying oven (Figure 1). The machine is designed for coating up to 120 cm wide polyester and plastic substrates. The plasma neutralizer is optionally used to negatively charge the polymethyl methacrylate panels (PMMA) surface in order to better prepare it to the coverage. Fully automated spraying is performed inside a ventilated chamber with the spray nozzles moving over the substrate. The four nozzles of each sprayer can be operated singly, in pairs or concomitantly. The spray nozzle (manufacturer and model are confidential) operated with 270 normal L/min air flow atomizing the coating suspension delivered at a flow rate of 200 mL/min per nozzle. After spraying, the substrate is dried in a drying oven. The spray chamber volume is about 6 m^3^ in volume with an inflow rate of about 3000 m^3^/h clean air and a bottom aspiration flow in order to maintain under pressure conditions inside the chamber. The air extracted from the spray chamber is cleaned by a M4 filter before being discharged into the atmosphere. No forced ventilation is present in the working area. The total dimension of the room containing the spray machine is about 6 × 15 m. Since the process is continuous, the cabin cannot be completely sealed because of the entrance and exit openings for the conveyor belt.

Six spray tests were carried out for each suspension (TiO_2_-N and AgHEC), combining the following three working parameters: suspension concentration, flow rate and substrate type (polyester fabric and PMMA). In total, 12 tests were performed. Table S1 details the spraying parameters of each test.

### 2.2. Methods

Measurements were taken simultaneously at the following three locations: inside the spray chamber, near the spray chamber at about 1 meter far from the spray nozzles (near field position—NF) and 6 meters from the spray chamber (far field position—FF). Particle numbers and mass concentrations were measured inside the spray chamber and at NF and FF positions in all 12 tests (see Table S1). Each test consisted of four sprays for a total test time length of ca. 40 minutes. Background concentrations (measured before and after each test) were subtracted from the measurements. Particle number concentrations, size distributions, lung deposited surface areas (LDSA) and mass concentrations were measured at the NF and FF at heights from 1 to 1.3 m corresponding to the level of the conveyor belt. The real time NF particle measurement position included the following:

- Particle mobility size distributions were obtained by Scanning Mobility Particle Sizer (SMPS), composed by a differential mobility analyzer (L-DMA mod. Grimm mod. 5400, Grimm Aerosol, Ainring, Germany), a condensation particle counter (CPC, Grimm mod. 5403, Grimm Aerosol, Ainring, Germany), and an X-ray soft charges neutralizer (TSI mod. 3088; Shoreview, MN, USA) instead of the original one based on ^241^Am (Grimm Mod. 5522). Nicosia et al. [24], applied a TSI soft X-Ray neutralizer to the Grimm L-DMA column obtaining a transfer function to correct the data. The SMPS scan time was ca 4.5 min with a 1.5 min retrace time. Mobility size was measured in the range from 10 nm to 1 μm.
- Particle optical size distributions were obtained by an optical particles counter (OPC Grimm mod. 1107 D, Grimm Aerosol, Ainring, Germany) in the 0.3–30 μm size range (in 32 channels) with a time resolution of 6 sec.
- LDSA concentrations (µm^2^/cm^3^) measured by a diffusion charger (Naneos Partector, Switzerland) in the size range from 10 to 400 nm. LDSA is a metric that it is correlated with the pulmonary deposition [4,5,25].
- Aerosol mass concentration was detected using an aerosol photometer (DustTrack mod. 8530, TSI Inc., Shoreview, MN, USA).

The real time FF particle measurement position included the following:

- Particle optical size distributions were obtained by optical particles counter (OPC Grimm mod. 1107 A, Grimm Aerosol, Ainring, Germany) in the 0.3–30 μm size range (in 32 channels) with a time resolution of 6 sec.
- LDSA concentrations (µm^2^/cm^3^) measured by a second diffusion charger (Naneos Partector, Switzerland) in the size range from 10 to 400 nm.

Inside the spray chamber, aerosol was measured by the following:

- Two low-cost optical particles counters SPS30 (Sensirion, Staefa, Switzerland) positioned at the left (SPS30_L) and at the right side of the spray nozzles (SPS30_R), respectively. SPS30 can measure number concentration (in the range 0–3000 p/cm^3^) of particles with diameter > 0.3 µm, in four dimensional classes: 0.5–1 µm; 1.0–2.5 µm; 2.5–4 µm; 4–10 µm.
- Aerosol mass concentration was detected by means of an aerosol photometer (DustTrack mod. 8520, TSI Inc., Shoreview, MN, USA).
- The UFP number concentration and the lung deposited surface area (LDSA) was obtained with a DiSCmini (Testo, Lenzkirch, Germany). Maximum detectable particle concentrations depends on particle size and averaging time. Typical value is 1·10^6^ p/cm^3^.

Off-line gravimetric PM samples were taken simultaneously inside the spray chamber and at NF by collecting the particles on absolute filters (PTFE, 1 µm porosity, Ø 47 mm) at 50 L/min flow rate (Bravo H-Plus, TCR Tecora, Italy). The mass concentrations were determined by weighing the filter before and after the sample collection (analytical balance, Mettler Toledo AX105).

In addition, filter samples (Nuclepore, porosity 0.22 µm) were collected for electron microscopy analysis by using a Field Emission Scanning Electron Microscopy (FESEM—Carl Zeiss Sigma NTS, Gmbh Öberkochen, Germany) coupled with an energy dispersive X-ray (EDX) micro-analyzer (EDS, mod. INCA Energy 300, Oxford instruments, UK). The FESEM samples were gold-coated (thickness = 5 nm).

Elemental analysis was performed by an ICP-OES 5100- vertical dual view apparatus (Agilent Technologies, Santa Clara, CA, USA) on the collected filters by a procedure reported in the Supporting Information.

## 3. Results and Discussion

### 3.1. Inside the Spray Chamber

**Figure 2:**
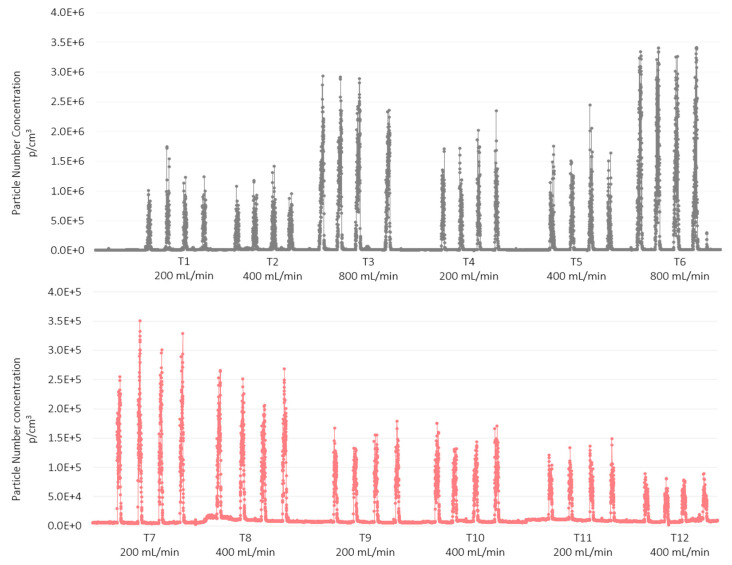
Particle number concentration measured inside the spray chamber with the DiSCmini. Size range: 0.03–0.4 µm- TiO_2_-N solution in grey; AgHEC in pink.

Figure 2 shows the UFP number concentration given by the DiSCmini for both nanomaterials (AgHEC and TiO_2_-N) sprayed on the two different substrates (polyester and PMMA). Each test comprises four sprays (four spikes in the figure). In the case of TiO_2_-N, particle number concentration is about one order of magnitude higher than for AgHEC. The increase in particle number concentration as a function of the number of operating nozzles was lower for the TiO_2_-N sprays (tests 1 to 3) compared to the AgHEC sprays.

**Figure 3:**
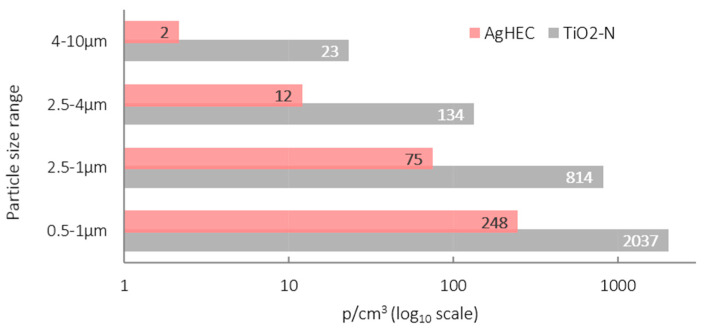
Histogram of the particle number concentration in different size classes measured in the spray chamber. Averaged SPS30 outputs. TiO_2_-N suspension (grey), AgHEC suspension (pink).

Particle size distribution inside the spray chamber, obtained with the SPS30 (4 size classes: 0.5–1 µm; 1.0–2.5 µm; 2.5–4 µm; 4–10 µm) is shown in Figure 3 for TiO_2_-N (grey) and for AgHEC (pink). The 0.5–1.0 µm size range of the UFPs concentration was observed to be lower for the TiO_2_-N suspension. Comparison between SPS30_L and SPS30_R showed a relative difference in particle number concentration of about 20% with one or four operating nozzles, while in the two-nozzle configuration, the concentration on the left side of the chamber was higher than on the right (about 70%).

### 3.2. Near Field and Far Field

**Figure 4:**
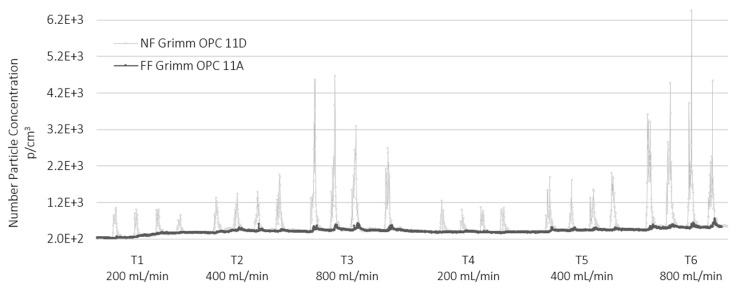
Comparison between particle number concentration of the TiO_2_-N suspension measured at the NF position (light grey line) and at the FF station (dark grey line). Particles size range 0.3–1 µm.

**Figure 5:**
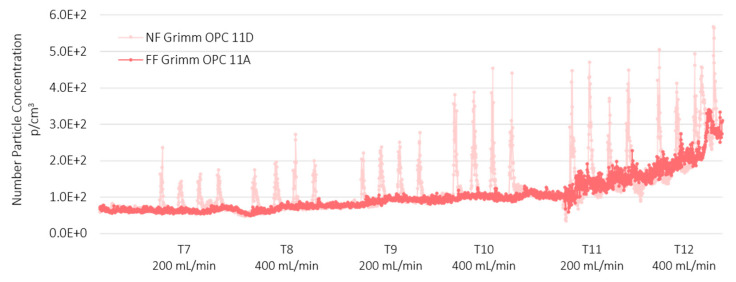
Comparison between particle number concentration of the AgHEC suspension at NF and FF position (light pink line) and at FF station (Red line). Particles size range 0.3–1 µm.

Figure 4 and Figure 5 show the data collected from the GRIMM OPCs (mod. 1107A in the FF and mod. 1107D in the NF position) for TiO_2_-N and AgHEC sprays, respectively.

The averaged particle number concentration measured using the SMPS at NF station was below 9·10^3^ p/cm^3^ (see Figure S3, Supporting Information) while in the size range 0.3–1 µm were below 4·10^2^ p/cm^3^. In general, the particle number concentration at FF was very low compared to the values measured at the NF position. In the case of TiO_2_-N (sprayed both on PMMA and polyester) the spray spikes were well correlated between the NF and FF stations. Sprays with AgHEC did not show spikes at the FF station. Figure 5 highlights the baseline increase observed in the afternoon tests with AgHEC, due to the exhaust emission from an engine outside the warehouse, which entered the room moving from the NF to the FF stations. The time shift between the maximum values recorded in the NF and FF positions was around 1 min which indicates a diffusion velocity of about 0.1 m/s (see Figures S1 and S2).

**Figure 6:**
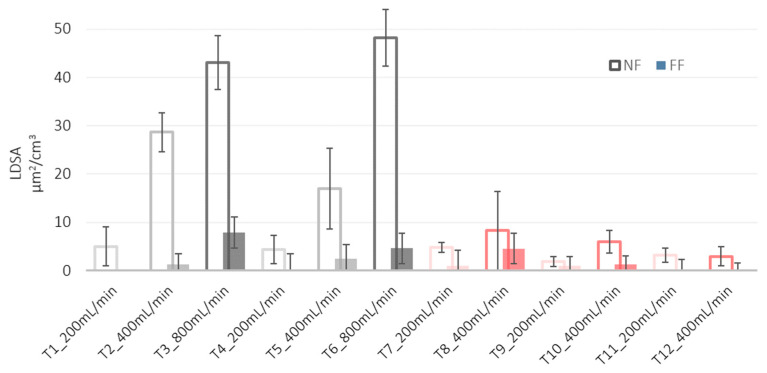
Histogram of LDSA of the particles emitted at the NF (empty bars) and FF (solid bars).

**Figure 7:**
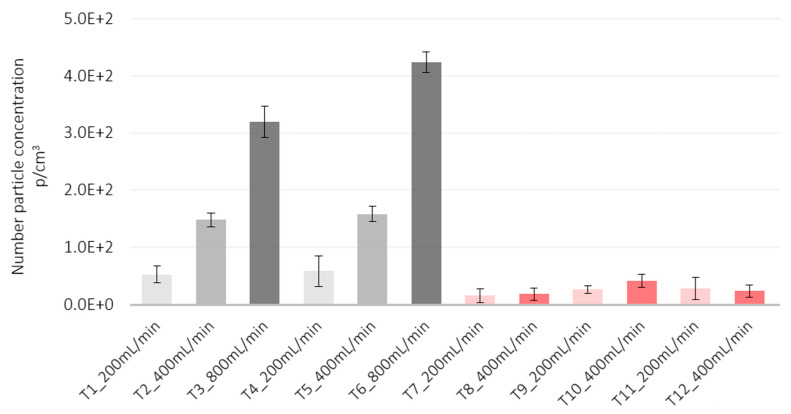
Histogram of particle number concentrations measured at the NF position. Particles size range 0.3–1µm. Grey bars TiO_2_-N; pink bars AgHEC.

Toxicological studies have shown that LDSA correlates with negative health effects [26]. In our experimental conditions, LDSA values were lower than 50 µm^2^/cm^3^ in all tests (see Figure 6) both at the NF and FF positions, and much lower than the peak value emitted by a burning candle (about 250 µm^2^/cm^3^) and comparable with urban background sites in Los Angeles and in Cassino [27]. The histogram of the particles number concentration measured by OPC at NF station and averaged over the whole test is given in Figure 7.

The particle number concentration of the TiO_2_-N suspension was slightly higher on polyester substrates (tests T4–T6) than on PMMA (tests T1–T3), a finding that could be due to the plasma beam turned on to better retain the sprayed material. With all four nozzles in operation, tests T3 and T6 had the highest flow rate and highest particle number concentration: 322 and 425 p/cm^3^ at T3 and T6 on PMMA and polyester, respectively.

**Figure 8:**
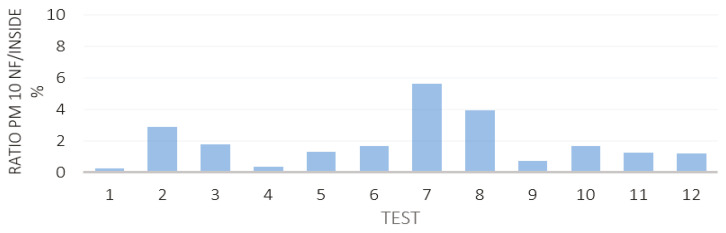
Percentage ratio between particle mass concentration measured at NF and inside the spray chamber using two DustTrak instruments.

Averaged particle number concentrations after subtraction of the background did not exceed 450 cm^3^ particles for both the suspensions (Figure 7), staying below the Nano Reference Values (NRVs) based of 2009 IFA benchmark levels [28] and dependent on particle density. For particle densities lower than 6 g/cm^3^, as in the case of TiO_2_-N, the proposed threshold concentration is 2·10^4^ p/cm^3^, while for higher particle densities, as in the case of AgHEC, the proposed threshold concentration is 4·10^4^ p/cm^3^. Figure 8 gives the percentage ratio between the particle mass concentration measured inside the spray chamber and at the NF position measured with two DustTrak, pointing out a released percentage below 4% for all the tests carried out (the average percentage ratios were 1.7% for TiO_2_-N and 1.5% for AgHEC).

These ratios give an indication of the containment capacity of the chamber. Table 1 shows the particle mass concentrations collected on the PTFE filters inside the spray chamber and a NF with their respective standard deviations. The uncertainty of the values was obtained by considering three standard deviations of ten blank filter weights. The ratio between the particle mass collected on the filters inside the spray chamber and at the NF position was 7.7% for TiO_2_-N, and 21.5% for AgHEC. The effective mass of the heavy metals Ti and Ag collected on the filters was calculated by ICP-OES (see SI for the analysis procedure). The percentage ratio between NF and inside chamber was 5.1% for Ti and 3.1% for Ag. Considering the ICP-OES results and the filter-based mass measurement, it was possible to estimate the percentage of the metal compared to the total amount of NPs collected.

**Figure 9:**
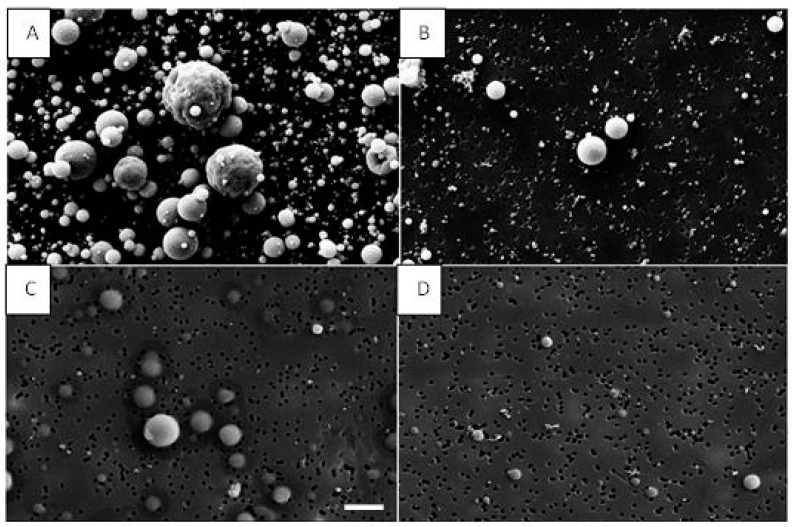
SEM images of particles collected on polycarbonate filters. (**A**) TiO_2_-N particles inside the spray chamber; (**B**) TiO_2_-N at the NF position; (**C**) AgHEC inside the spray chamber; (**D**) AgHEC at NF station. Scale bar (picture C) is 2 µm and all images are in the same scale.

Figure 9 shows SEM images of particles from both used suspensions inside the chamber and at the NF position. Lower particle number concentrations were observed at NF for both AgHEC and TiO_2_-N suspensions compared inside the chamber. The images also confirm the wide NP dispersion after the spray process.

**Figure 10:**
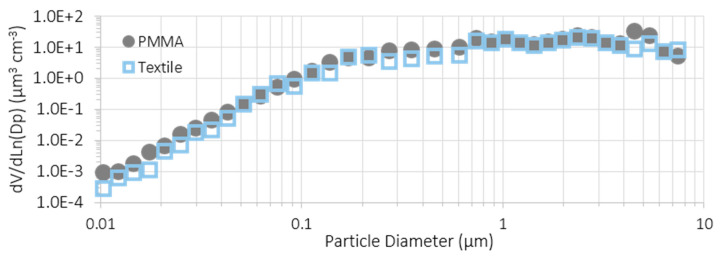
Volume aerosol size distributions for TiO_2_-N sprays over different substrates in the NF station.

Figure 10 shows an example of the aerosol volume size distribution obtained with TiO_2_-N spray coating tests on PMMA and polyester at the NF station. Merging the outputs from the SMPS and OPC for each test, volume particle size distributions were obtained in the range from 0.01 to 30 μm. We merged the aerosol size distributions from the SMPS and from the OPC by averaging the overlapping size intervals from 0.74 µm to 0.87 µm size bins: SMPS data were used for lower sizes and OPC data for higher sizes. It was assumed that mobility and optical particle diameters were the same. These distributions will be used as an input for inhalation dose models. Both volume size distributions are consistent with an important contribution from the fine size fractions. Volume aerosol concentration is mainly affected by aggregated particles (as showed in Figure 9).

### 3.3. Comparison of Substrates and Suspensions

**Figure 11:**
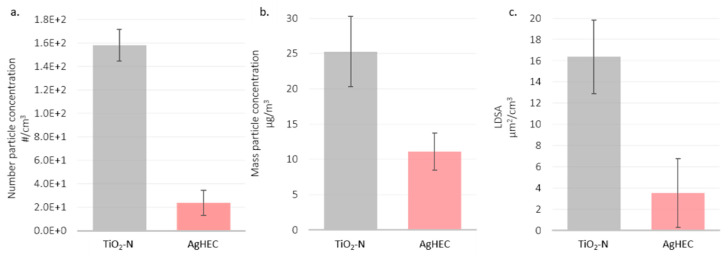
Histograms of T5 and T12 compared in the NF station: (**a**) particle number concentration in the particle size range 0.3–1µm, (**b**) mass particle concentration in the particle size range > 0.1 µm, (**c**) LDSA values in the particle size range 0.03–0.4 µm. TiO_2_-N in grey, AgHEC in pink.

The effect of the sprayed materials for the same substrate (polyester) and flow rate (400 mL/min) was evidenced by comparing test 5 (T5) with test 12 (T12): TiO_2_-N, 1% *w*/*w* and AgHEC 0.1% *w*/*w*. Figure 11 shows particle number, mass and LDSA concentrations measured at the NF station for both tests.

**Figure 12:**
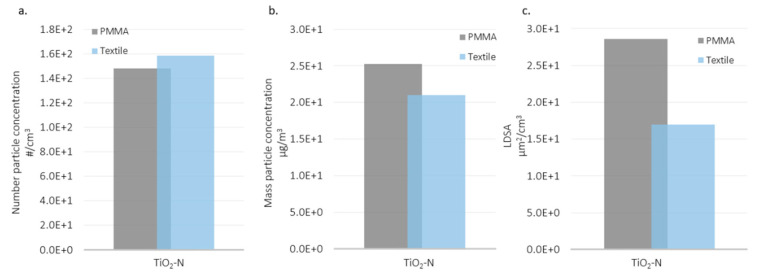
Histograms comparing different substrates sprayed with TiO_2_-N in the NF station: (**a**) particle number concentration in the particle size range 0.25–1µm, (**b**) mass particle concentration in the particle size range >0.1 µm, and (**c**) LDSA values in the particle size range 0.03–0.4 µm.

Spraying the TiO_2_-N suspension released aerosol concentrations one order of magnitude higher than the AgHEC suspension. The averaged particle mass concentrations for the TiO_2_-N suspension were almost double the emissions released by the silver suspension. The same result was found for LDSA concentrations. We suggest that AgHEC solvent water droplets, which evaporate more slowly than TiO_2_-N solvent ethanol droplets determine higher losses inside the spray chamber. Figure 12 shows the results collected for TiO_2_-N applied to the two different substrates (T2 and T5) (TiO_2_-N, 1% in ethanol with 400 mL/min flow rate). In the case of the TiO_2_-N suspension, the released particle number concentration was comparable for both the PMMA and polyester substrates. For TiO_2_-N suspension the released particles number and mass concentrations were quite comparable using PMMA or polyester as substrates. LDSA concentrations showed higher values when PMMA was used respect to polyester. This could be justified by the higher absorbing capacity of polyester than PMMA together with the released ions from the plasma neutralizer. 

## 4. Conclusions

An industrial spray-coating process was monitored with real time and off-line techniques inside a spray chamber and at NF and FF positions. Two NPs suspensions (TiO_2_-N 1% *w*/*w* in ethanol, and AgHEC in water at different concentrations) were sprayed using a pneumatic atomizer over the following two different substrates: PMMA and polyester. Tests were carried out at various machine parameters to take into account best- and worst-case scenarios. In general, TiO_2_-N sprays showed higher particle release than AgHEC sprays. While atomized droplets should evaporate rapidly in the case of the ethanol-based TiO_2_-N spray, this might not be the case for the water-based AgHEC suspension, which could cause a higher particle capture for this latter inside the spray chamber. NF and spray chamber particle mass concentration ratios were below 5% as measured by aerosol photometers and ICP-OES elemental analysis both for the AgHEC and TiO_2_-N sprays. The FF results showed a correlation with the NF spikes (single sprays) only at the highest spray TiO_2_-N suspension flow rate. In the best scenario case, single-spike spray particle number concentrations for TiO_2_-N measured with the OPC was about 10^3^ p/cm^3^ for PMMA and polyester, while the worst-case scenario registered 5·10^3^ p/cm^3^ and 6.5·10^3^ p/cm^3^ for PMMA and polyester, respectively. AgHEC particles release was one order of magnitude lower for both the substrates. In general, the particle number concentration values measured by means of SMPS at NF station were lower than the particle number concentrations measured with other spraying techniques [13]. In addition, compared with values reported by IFA in 2009 for these particle densities, the emissions we found were all below the benchmark levels [29].

NIOSH recommends airborne exposure limits of 2400 µg/m^3^ for fine TiO_2_ [30] and 10 µg/m^3^ for Ag based NPs [31] values far above those measured in the field monitoring campaign measured both by DustTrak and especially by ICP-OES mass analysis. LDSA at NF was below 50 µm^2^/cm^3^ for all tests, a value that is comparable or lower than urban background sites in Los Angeles or Cassino (Italy) [27].

To the best of our knowledge, this is one of the few studies dealing with the characterization of nanoparticles emitted from a continuous pneumatic spray coating process at industrial scale. Our results could be generalized to other similar spray cabins (continuous spray processes).

## Figures and Tables

**Table 1:** Aerosol mass concentrations obtained by gravimetric measurement, and ICP-OES mass analysis.

Material	Inside Spray Chamber (µg/m^3^)	NF (µg/m^3^)	Ratio (%)
TiO_2_-N ^a^	1198 ± 2	93 ± 6	7.7
Ti ^b^	491 ± 4	24.7 ± 0.6	5.1
AgHEC ^a^	172 ± 5	37 ± 6	21.5
Ag ^b^	13.2 ± 0.3	0.35 ± 0.03	3.1

^a^ Filter-based gravimetric measurements ^b^ ICP-OES analysis.

## Data Availability

The data is available on reasonable request from the corresponding author.

## Supplementary Materials

The following are available online at www.mdpi.com/2079-4991/12/3/313/s1, Table S1. Summary of the spraying parameters adopted for each test in terms of: kind of nanomaterial, number of nozzles deployed during the spray test, total flow rate, coated substrate and consumed material, Table S2. Instrument position and characteristics, Table S3. Heating program for acid digestion of the filter samples, Table S4. Results from ICP-OES, Figure S1. Experimental setup: inside the spray chamber (above), NF (middle) and FF (bottom), Figure S2. Record of aerosol particle number concentrations coming from an external source, Figure S3. Averaged particle number concentration versus tests. NF measurements.
