# Supplementary material for: Particles Emission from an Industrial Spray Coating Process Using Nano-Materials
Source: Nanomaterials (Basel). 2022 Jan 18;12(3):313. doi: 10.3390/nano12030313 (PMC8838285; doi:10.3390/nano12030313)
Supplement: Supplementary file 1 [file nanomaterials-12-00313-s001.zip › nanomaterials-1451682-supplementary.pdf]

nanomaterials

Supplementary Materials

Particles Emission from an Industrial Spray Coating Process Using Nano-Materials

Benedetta Del Secco [1,*], Sara Trabucco [1], Fabrizio Ravegnani [1], Joonas Koivisto [2], Magda Blosi [3], Simona Ortelli [3] Marko Altin [4], Gianni Bartolini [4], Anna Luisa Costa [3] and Franco Belosi [1]

1 National Research Council, Institute of Atmospheric Sciences and Climate (CNR-ISAC), Via P. Gobetti 101, 40129 Bologna, Italy, b.delsecco@isac.cnr.it
2 Air Pollution Management APM, Mattilanmäki 38, 33610 Tampere, Finland
3 National Research Council of Italy-Institute of Science and Technology for Ceramics (ISTEC-CNR), Via Granarolo 64, I-48018 Faenza (RA), Italy
4 Wiva Group srl, Via Siena 47, 50142 Firenze (FI), Italy
* Correspondence: b.delsecco@isac.cnr.it

1. Test descriptions and instrument characteristics

Table S1. Summary of the spraying parameters adopted for each test in terms of: kind of nanomaterial, number of nozzles deployed during the spray test, total flow rate, coated substrate and consumed material.

Test	Material	Number of working Nozzles	Flow rate (mL/min)	Substrate	Material consumed (Kg)
Test 1	TiO_2-N	1	200	PMMA	1.03
Test 2	TiO_2-N	2	400	PMMA	1.25
Test 3	TiO_2-N	4	800	PMMA	1.58
Test 4	TiO_2-N	1	200	Textile	1.04
Test 5	TiO_2-N	2	400	Textile	1.29
Test 6	TiO_2-N	4	800	Textile	1.65
Test 7	AgHEC (0.01%)	1	200	Textile	1.24
Test 8	AgHEC (0.01%)	2	400	Textile	1.39
Test 9	AgHEC (0.05%)	1	200	Textile	1.16
Test 10	AgHEC (0.05%)	2	400	Textile	1.40
Test 11	AgHEC (0.1%)	1	200	Textile	1.17
Test 12	AgHEC (0.1%)	2	400	Textile	1.41

Table S2. Instrument position and characteristics.

Instrument	Measured metric	Size range	Measurement location	Manufacturer, model
		**Online instruments**		
**Diffusion charger**	N, SA, D_P	30 nm - ~700 nm	IN/NF/FF	IN 1× DISCMini, Testo<br>NF/FF 2× Partector, Naneos
**OPC**	N, PSD<br>(optical sizer)	250 nm - 20 μm	NF/FF	1× Model 11-D, Grimm<br>1× Model 11-A, Grimm
**OPC**	N, PSD<br>(optical sizer)	500 nm - 10 μm	IN	2× Sensirion Mod. SPS30
**SMPS**	N, PSD<br>(mobility sizer)	11.1 nm - 1082 nm	NF	1× Series 5.400 with long-DMA, Grimm
**DustTrack**	PM<br>(photometer)	> 0.01 mg m^{-3}	IN/NF	2 DustTrack I and II (TSI)
		**Offline instruments**		
Direct collection	Total dust<br>(Teflon, Millipore, 0.45 μm)		IN/NF	4 pumps:<br>2× Bravo, Hplus (TCR, Tecora);<br>1× DualPump(XearPro)<br>1× Zambelli (Explorer)
Electron microscopy samples	Total dust<br>(Polycarbonate, Nuclepore 0.2 μm)		NF	Pump (Zambelli)

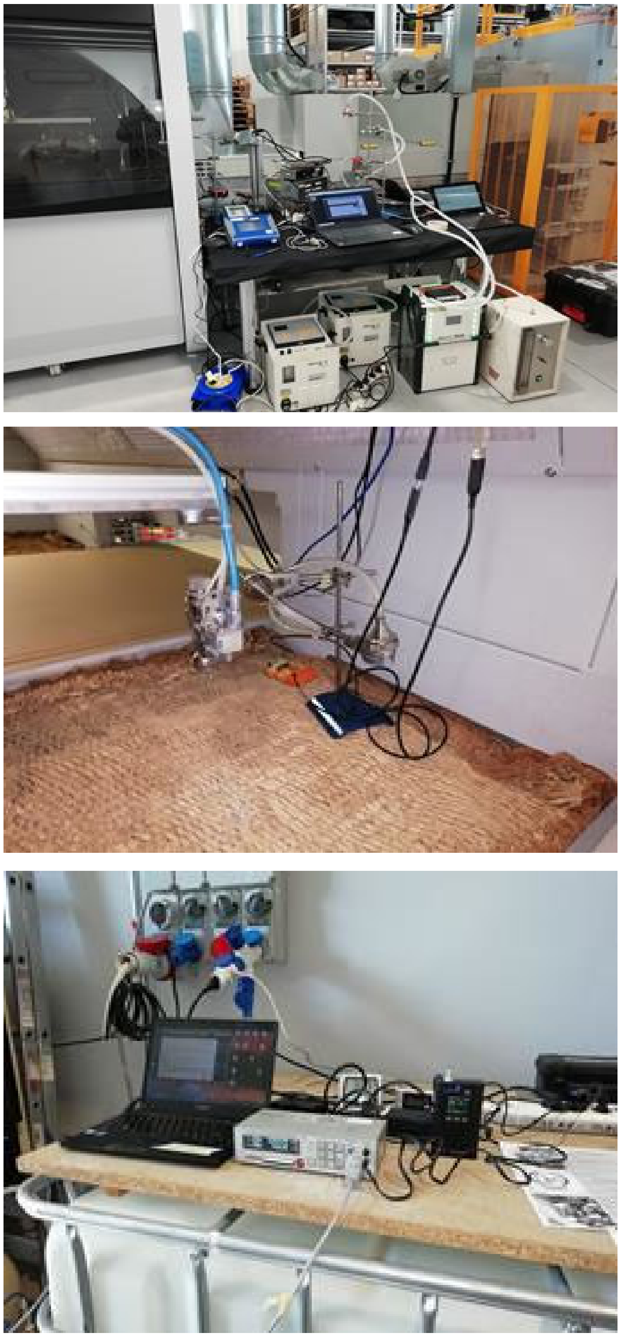

Figure S1. Experimental setup: inside the spray chamber (above), NF (middle) and FF (bottom).

2. Indoor contamination

Starting from 1:15 pm the aerosol particle concentration inside the warehouse (where the spray machine was located) started to increase due to an external source (engine combustion). The contaminant aerosol front arrived at the NF station and moved towards the FF. Figure S2 shows the spike recorded at NF station (blue one) and after about one

minute the recorded spike at the FF station (red curve). Measurements were obtained by means of the OPCs 11-D and 11-A.

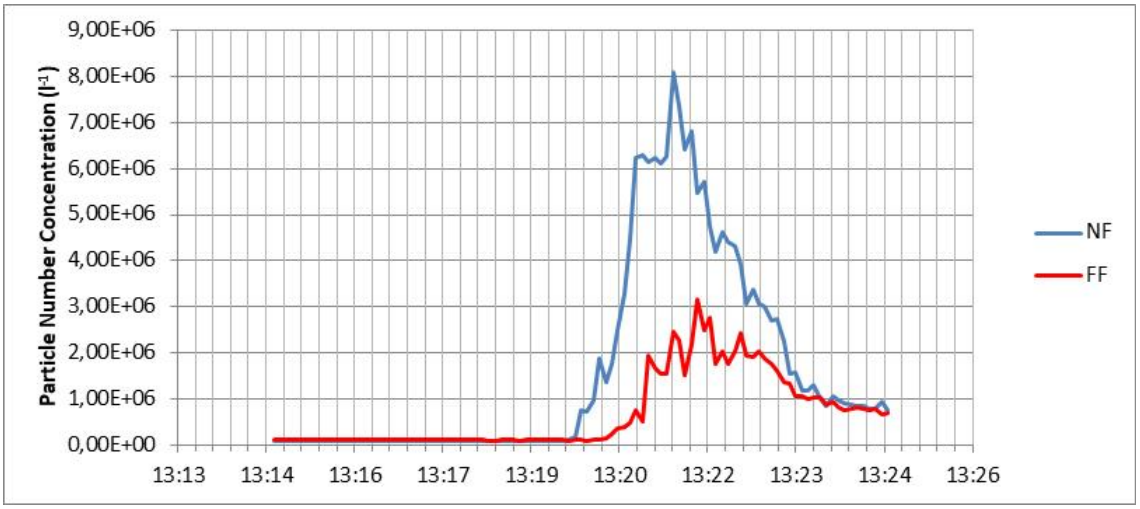

Figure S2. Record of aerosol particle number concentrations coming from an external source.

In Figure S3 are reported the averaged particles number concentration for each test

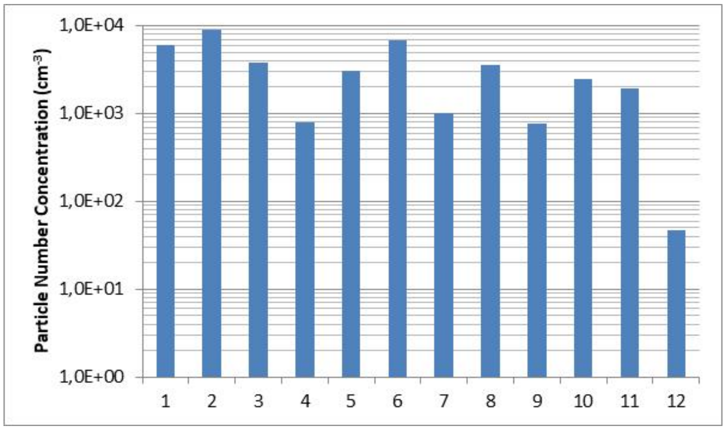

Figure S3. Averaged particle number concentration versus tests. NF measurements.

3. ICP-OES analysis

Digestive procedure

The filters were acid treated by microwaves digestion. The acid mixture used for samples mineralization was composed by HNO_3 (65%) and H_2SO_4 (96%), in a 1:1 ratio. The acid mixture (3 mL) was added in Teflon tubes with ca. 0.17 g of the filter samples were added to the digestive solution. The microwave system EM-45/A Milestone was used to digest the samples following the procedure reported in Table 1B. Afterwards, the samples were allowed to cool down for 30 minutes in vent mode at room temperature. The obtained solutions were diluted to 10 mL and analyzed without another treatment.

Table S3. Heating program for acid digestion of the filter samples.

Stage	Ramp Time (min)	Power (Watt)
1	1	250
2	1	0
3	4	250
4	4	400
5	4	600

Elemental analysis

All digested samples were analysed by an ICP-OES 5100—vertical dual view apparatus (Agilent Technologies, Santa Clara, CA, USA), coupled with OneNeb nebulizer and equipped with an Autosampler. The analysis was performed in radial viewing mode, and calibration curves were obtained with 0.05, 0.10, 1.00, 10.00 and 100.00 mg L-1 standards for Ag and Ti element. Nitric acid (65%) and sulfuric acid (96%) were added to standards as in the digested samples. Calibration curves were evaluated and showed a good correlation, coefficient (R2) above 0.99. Results from ICP-OES were reported as the average of three independent measurements with relative standard deviation (RSD) %.

Table S4. Results from ICP-OES.

Samples Teflon Filter	VOLUME PUMP (m^3)	Ti mass (mg)	Ag mass (mg)
background INSIDE 15/02	12.130	0.002	0.000
INSIDE TiO_2-N 16/02	13.930	6.840	0.000
INSIDE AgHEC 17/3	13.920	0.005	0.184
background NF 15/02	10.171	0.002	0.000
NF TiO_2-N 16/2	12.388	0.304	0.000
NF AgHEC 17/2	11.322	0.006	0.004
